# Mental health problems among secondary school students 10-24 years in Kilimanjaro region, Northern Tanzania

**DOI:** 10.4314/ahs.v25i4.20

**Published:** 2025-12

**Authors:** Jackline T Shirima, James Samwel Ngocho, Lisbeth Mhando, Rehema A Mavura, Innocent B Mboya

**Affiliations:** 1 Department of Epidemiology and Applied Biostatistics, Institute of Public Health, Kilimanjaro Christian Medical University College, Moshi, Tanzania; 2 Department of Community Health, Kilimanjaro Christian Medical Center, Moshi, Tanzania; 3 Department of Behavioral and Social Sciences, Institute of Public Health, Kilimanjaro Christian Medical University College, Moshi, Tanzania; 4 Department of Translational Medicine, Lund University, Malmö, Sweden

**Keywords:** mental health problems, adolescents, young people, Global School-based Student Health Survey, Regional School-based Student Health Survey, Kilimanjaro, Tanzania

## Abstract

**Background:**

Three-quarters of all mental health problems begins between 10-24 years. When not treated, adolescents and young people with mental health problems are at high risk of abuse, suicide, and substance use, which have long-term consequences that negatively impact physical, and economic productivity. The study aimed to determine the prevalence of mental health problems among secondary school students 10-24 years in the Kilimanjaro region, northern Tanzania.

**Methodology:**

We utilized secondary data from two repeated cross-sectional surveys conducted in 2019 and 2022 among students aged 10-24 years in the Kilimanjaro region, northern Tanzania. A chi-square test was used to compare mental health problem proportions by survey year and other participant characteristics. Multivariable logistic regression estimated odds ratios and 95% confidence interval to determine factors associated with mental health problems.

**Results:**

The median age of 4955 study participants was 15 (14, 17), 64% were 15-19 years, 53.9% were females, and 65% participated in survey 1. The overall prevalence of mental health problems was 29.2% (survey 1; 27.4% and survey 2; 32.6%). Overall, higher odds of mental health problems were among students aged 20-24 years than those aged 10-14 years, among females, currently using any substances, ever had sex, ever been physically attacked, ever been bullied, and those ever-missed classes.

**Conclusion:**

Mental health problems are highly prevalent among secondary school adolescents and young people in the Kilimanjaro region and were common among those aged 20-24 years, females, final year students, current substance users, history of having sex, ever missed classes, and being bullied. In-school programs for mental health issues awareness among students should be improved.

## Introduction

Mental health is defined by the World Health Organization (WHO) as a state of well-being in which an individual recognizes his or her abilities, can cope with normal life stresses, can work productively, and fruitfully, and can contribute to his or her community^1^. Half of all mental health problems in adults begin before 14 years and 75% by age 24 years, and if not treated early enough, can harm the lives of both adults and children^2^,^3^. In a systematic review and meta-analysis, the estimated prevalence of mental disorders in the general population globally was 29.1%^4^, and 13% among adolescents^5^.

A systematic review in 16 sub-Saharan African countries (SSA) reported the common mental health disorders and symptoms in adolescents being depression (26.9%), anxiety (29.8%), emotional and behavioral problems (40.8%), stress (21.5%), and suicidal ideation (20.8%)^6^. In Tanzania, the 2014 Global School-based Student Health Survey (GSHS) report indicated that students felt lonely always or most of the time (7%), felt worried and couldn't sleep at night (6.4%), seriously considered suicide (13.9%), made a suicide plan (9.5%), and attempted suicide (11.5%)^7^. Few available studies on young people and adolescent mental health (AMH) in the Kilimanjaro region indicate a high prevalence of mental health problems (33% and 13.7%, respectively)^8^,^9^.

The most common risk factors for mental health problems include age (common among those aged 10-24 years), sex (female than males), ethnicity, parental distress, poor general health, being in the fourth secondary school year, living in a rural area, smoking or drinking alcohol, and engaging in sexual behavior^10^,^11^,^12^. Adolescents and young people with mental health problems are more likely to participate in risky activities including unprotected sexual activities, substance abuse, and unintentional injuries, which result in school dropout, bullying, and suicide^13^.

The Sustainable Development Goals include efforts to prevent, treat, and promote mental health and well-being to minimize one-third of premature mortality from Non-Communicable Diseases^14^. In Tanzania, a school mental health literacy (MHL) curriculum resource training approach has been established to increase teachers' MHL (improved knowledge, decreased stigma, and positive help-seeking efficacy), hence improving mental health outcomes for students^15^. GSHS provides essential data on adolescent and young people's risk behaviors and is resourceful in determining the burden of mental health problems to inform programs for improving school and adolescent health^7^. This study aimed to determine the prevalence of mental health problems, compare the prevalence across two survey years (2019 and 2022), and determine associated factors among secondary school students 10-24 years in the Kilimanjaro region, northern Tanzania.

## Methodology

### Data source

Data for this study came from two repeated cross-sectional regional school health surveys (RSHS) that assessed the risk behaviors of secondary school adolescents and young people in four districts of the Kilimanjaro region. The surveys were conducted in 2019 (among form one students) and 2022 (among form four students) by the Institute of Public Health (IPH) of Kilimanjaro Christian Medical College (KCMUCo). The surveys were part of health promotion training activities for the Doctorate of Medicine students at the College. The methodology of the 2019 survey has also been described elsewhere^16^,^17^.

### Study population

The study population was students aged 10-19 years for survey 1 and 13-24 years for survey 2 from the same class attending public secondary schools in the Kilimanjaro region. The study included only adolescents and young people who attended school on the day of data collection and provided assent or informed consent. Those whose ages and sex were not specified in the dataset, above 19 years of age for the 2019 survey, and those who did not respond to any of the questions on mental health problems were excluded. In survey 1; 3,227 adolescents participated in the study, and 1,742 in survey 2. The number is less in survey two because the IPH had less funding to implement the study, hence a total of 4,969 students in the two surveys. After exclusions, 4,955 students were analyzed (survey 1, 65% and survey 2, 35%).

### Data collection

Data for both surveys were collected using a self-administered questionnaire from the Regional School-based Student Health Survey (RSHS). The tool has undergone validation and has been applied in numerous similar surveys across Low and Middle-Income Countries including Tanzania^18^. The RSHS was adopted from the GSHS developed by WHO/CDC to help countries measure and assess the behavioral risk and protective factors among students aged 13-17 years^5^. The Standard English Questionnaire from WHO was produced, translated into Swahili, pre-tested, and used in Tanzania GSHS^18^. Data collected during the survey include basic demographic characteristics (age, sex, and district), violence, unintentional injury, dietary behaviors, hygiene, mental health, physical activity, substance use, and sexual behaviors. Trained medical students from KCMUCo collected the data, whose main role was to describe the study purpose to the eligible participants, responded to all their questions, guide them to respond to each question systematically, and ensure completeness of the questionnaires.

### Study variables and measures

The outcome variable in this study was mental health problems, defined as experiencing any mental disorders characterized by changes in mood, perception, and behavior causing distress, such as loneliness, worry, sleep problems, feeling attacked, and suicide (ideation, plan, and attempt)^1^,^7^,^18^. Participants who reported experiencing any of these symptoms or behaviors during the past 12 months were considered as having mental health problems^7^. The responses to questions related to loneliness, worry, sleeping problems, emotional changes, and feeling attacked were on a Likert scale of never, rarely, sometimes, most of the time, and always. The response was dichotomized as ‘yes’ if most of the time/always, and ‘no’, if never, rarely, or sometimes. Students were considered to attempt suicide if they reported an attempt at least once during the past 12 months.

The explanatory variables included socio-demographic and behavioral characteristics and social and psychological factors. Socio-demographic characteristics included age in years, sex (male, female), schooling district (Moshi municipality, Moshi rural, Siha, and Hai districts), and survey year (2019, 2022). Behavioral characteristics included ever being physically attacked (none, 1+ times); ever engaged in a physical fight; ever being bullied; ever being seriously injured; currently using any substance ‘Yes’ if used any of the substances (alcohol, cigarette, tobacco, recreational drugs such as cocaine, and heroin, marijuana, khat, amphetamines) in the past 30 days and ‘No’ if otherwise; and ever had sex. Social and psychological characteristics were, ever missed classes or school (none, 1+ days); Number of close friends (none, 1+ friends); parents or guardians checking participant's homework, understanding participants' problems or worries, knowing what participants were doing in their free time (never, rarely, sometimes, most of the time; always). These measures have been used in several similar studies in LMICs and are reported to have good validity^7^,^17^,^18^,^19^.

### Data analysis

Data were cleaned and analyzed using SPSS software version 20. Categorical variables were summarized using frequencies and percentages whereas continuous variables were summarized using median and inter-quartile range. The chi-square test was used to compare mental health problem proportions by survey year and other participant characteristics. Multivariable logistic regression analysis was used to estimate odds ratios (OR) and 95% confidence intervals (CI) to determine factors associated with mental health problems. The unadjusted logistic regression analysis models determined the association between the independent variables and mental health problems (binary variable). For the adjusted logistic regression analysis, variables that had the likelihood ratio p-values of < 0.1 in the unadjusted analysis were included in the adjusted analysis. Statistically significant associations were declared at a 5% threshold level.

## Results

### Participant socio-demographic characteristics

Among 4,955 secondary school students who participated in this study, the median age was 15 years, with an interquartile range of 14-17. Sixty-four percent were 15-19 years old, females (53.9%), were schooled in Moshi district (37%), Moshi municipal (24.6%), Siha district (22.6%), and Hai district (15.8%). Survey 1 contributed most (65%) of the participants in this study (Table 1).

**Table 1:** Socio-demographic characteristics of secondary school students in the Kilimanjaro region (N=4955)

	Overall	Survey 1	Survey 2	P-value
Variables	n (%)	n (%)	n (%)	
**Age (years)**				<0.001
10-14	1729 (34.9)	1726 (53.5)	3 (0.2)	
15-19	3169 (64.0)	1498 (46.5)	1671 (96.5)	
20-24	57 (1.2)	0	57 (3.3)	
Mean (SD)	15 (14, 17)*	14 (1.14)	17 (1.07)	
**Sex**				0.099
Male	2286 (46.1)	1515 (47.0)	771 (44.5)	
Female	2669 (53.9)	1709 (53.0)	960 (55.5)	
Schooling district**				<0.001
Moshi municipality	1203 (24.6)	667 (20.7)	536 (32.1)	
Moshi district council	1814 (37.1)	1342 (41.6)	472 (28.2)	
Hai district council	772 (15.8)	533 (16.5)	239 (14.3)	
Siha district council	1106 (22.6)	682 (21.2)	424 (25.4)	

**Survey year (Total)**		3224 (65.0)	1731 (35.0)	

SD = Standard Deviation

* Median (interquartile range)

** Frequency do not tally due to missing values

### Social and behavioral characteristics of participants

Among participants in the study, 37% reported the current use of at least one substance, ever had sex (10.6%), and less than a quarter (23.5%) reported ever missing classes or school without permission. Twenty-two percent of the students reported ever being physically attacked while 18.5% ever engaged in physical fights. Students who reported ever being seriously injured were 17.1%, and 11.4% had ever been bullied. Over two-thirds (68.3%) of the students said their parents or guardians ever checked their homework, and 47.7% said their parents or guardian ever knew what they do in their free time (Table 2).

**Table 2:** Social and behavioral characteristics of secondary school students in Kilimanjaro region (N=4955)

Variables	Overall n (%)	Survey 1 n (%)	Survey 2 n (%)	P-value
Current substances use*†				<0.001
No	1176 (63.0)	374 (47.2)	802 (74.5)	
Yes	692 (37.0)	418 (52.8)	274 (25.5)	
Ever had sex*				<0.001
No	4419 (89.4)	2923 (90.7)	1496 (86.9)	
Yes	525 (10.6)	300 (9.3)	225 (13.1)	
Ever missed classes or school*				<0.001
No	3773 (76.5)	2608 (80.9)	1165 (68.0)	
Yes	1162 (23.5)	615 (19.1)	547 (32.0)	
Number of close friends*				0.305
No friends	425 (8.7)	288 (9.0)	137 (8.1)	
>1 friend	4483 (91.3)	2927 (91.0)	1556 (91.9)	
Ever been physically attacked*				<0.001
No	3839 (77.7)	2444 (75.8)	1395 (81.3)	
Yes	1099 (22.3)	779 (24.2)	320 (18.7)	
Ever engaged in a physical fight*				<0.001
No	4033 (81.5)	2486 (77.1)	1547 (89.7)	
Yes	916 (18.5)	738 (22.9)	178 (10.3)	
Ever been seriously injured*				<0.001
No	4104 (82.9)	2584 (80.1)	1520 (88.1)	
Yes	846 (17.1)	640 (19.9)	206 (11.9)	
Ever been bullied*				<0.001
No	4380 (88.6)	2785 (86.4)	1595 (92.7)	
Yes	564 (11.4)	439 (13.6)	125 (7.3)	
Parent/guardian ever checked your homework*				0.031
			
No	1567 (31.7)	988 (30.7)	579 (33.7)	
Yes	3375 (68.3)	2234 (69.3)	1141 (66.3)	
Parent/guardian ever understood your problems*				0.624
			
No	2575 (52.1)	1686 (52.3)	889 (51.6)	
Yes	2370 (47.9)	1536 (47.7)	834 (48.4)	
Parent/guardian ever knew what you do on free time*				<0.001
			
			
No	2588 (52.3)	1612 (50.0)	976 (56.5)	
Yes	2361 (47.7)	1611 (50.0)	750 (43.5)	

* Frequency does not tally due to missing values

† Using at least one substance (alcohol, cigarette, tobacco, khat, marijuana, and amphetamines) in the past 30 days

### Prevalence of mental health problems among secondary school adolescents

The overall prevalence of mental health problems for the two surveys was 29.2% (survey 1; 27.4% and survey 2; 32.6%), (p<0.001). Overall, 8.5% of the students reported to have ever felt lonely, 11.3% ever been worried, 4.5% ever lacked sleep, 6.9% ever considered suicide, 4.5% ever planned suicide, and 3.5% ever attempted suicide. Four percent of all the students had ever experienced emotional changes, 5.6% ever had auditory hallucinations, 4.6% ever had visual hallucinations, and 1.7% ever felt harmed or killed. These proportions were comparable between the two survey years (Table 3).

**Table 3:** Prevalence of mental health problems by survey year of secondary school students in Kilimanjaro region (N= 4955)

Variables	Overall (%)	Survey 1 n (%)	Survey 2 n (%)	P-value
Ever felt lonely*				
No	91.5	3004 (93.2)	1528 (88.3)	<0.001
Yes	8.5	220 (6.8)	202 (11.7)	
Ever been worried*				
No	88.7	2834 (90.6)	1472 (85.3)	<0.001
Yes	11.3	293 (9.4)	253 (14.7)	
Ever lack sleep*				
No	95.5	3094 (96.0)	1634 (94.6)	0.023
Yes	4.5	130 (4.0)	94 (5.4)	
Ever experience emotional changes*				
No	95.9	3043 (95.8)	1655 (96.1)	0.652
Yes	4.1	132 (4.2)	67 (3.9)	
Ever had auditory hallucinations*				
No	94.3	3038 (94.2)	1636 (94.7)	0.466
Yes	5.6	186 (5.8)	91 (5.3)	
Ever had visual hallucinations*				
No	95.4	3072 (95.3)	1649 (95.6)	0.591
Yes	4.6	151 (4.7)	75 (4.4)	
Ever felt someone wanted to harm you*				
No	98.3	3166 (98.2)	1701 (98.4)	0.593
Yes	1.7	57 (1.8)	27 (1.6)	
Ever considered suicide*				
No	93.1	3024 (93.8)	1580 (91.8)	0.007
Yes	6.9	200 (6.2)	142 (8.2)	
Ever planned suicide*				
No	95.5	3096 (96.0)	1624 (94.5)	0.015
Yes	4.5	128 (4.0)	94 (5.5)	
Ever attempted suicide*				
No	96.5	3128 (97.0)	1641 (95.6)	0.008
Yes	3.5	96 (3.0)	76 (4.4)	
Mental health problems (overall) ^†^*				
No	70.8	2257 (72.6)	1145 (67.4)	<0.001
Yes	29.2	851 (27.4)	555 (32.6)	

* Frequency does not tally due to missing values

† Proportion of students who experienced at least one mental health problem

### Factors associated with mental health problems

Table 4 shows the results of the adjusted analysis by each survey year and in general. Age 10-19 years (AOR=0.37, 95% CI: 0.15- 0.92), and parent or guardian knew what students did in free time (AOR=0.61, 95% CI: 0.42-0.89) which had lower odds of mental health problems compared to their equals, and ever had sex (AOR=1.75, 95% CI: 1.12-2.72) which had increased odds of mental health problems compared to never had sex, were significant factors for mental health problems in survey 2 but not in survey 1. In survey 2, adolescent students had lower odds of mental health problems compared to young people (AOR=0.37, 95% CI: 0.15- 0.92). Female sex (AOR=1.56, 95% CI: 1.08-2.26), current substance use (AOR=3.00, 95% CI: 1.87-4.81), and parent or guardian ever checked students' homework (AOR=1.54, 95% CI: 1.05-2.26) were significant positive factors for mental health in survey 1 but not in survey 2. Ever been physically attacked had lower odds of mental health problems, and ever bullied had higher odds of mental health problems compared to their counterparts in both surveys.

**Table 4:** Factors associated with mental health problems stratified by survey year among Secondary school students in the Kilimanjaro region

Variables	Overall AOR (95% CI)	Survey 1 AOR (95% CI)	Survey 2 AOR (95% CI)
**Age (years)**			
10-14	1	1	-
15-19	1.07 (0.82, 1.38)	0.93 (0.64, 1.34)	-
10-19*	-	-	0.37 (0.15, 0.92)
20-24	4.41 (1.96, 9.90)	-	1
**Sex**			
Male	1	1	1
Female	1.40 (1.12, 1.74)	1.56 (1.08, 2.26)	1.40 (0.98, 1.99)
**Schooling district**			
Moshi MC	1	1	1
Moshi DC	0.64 (0.50, 0.83)	0.56 (0.34, 0.94)	0.76 (0.52, 1.12)
Hai DC	0.62 (0.44, 0.88)	0.71 (0.38, 1.35)	1.03 (0.37, 2.84)
Siha DC	0.68 (0.50, 0.94)	0.50 (0.26, 0.97)	0.73 (0.46, 1.13)
**Current substance use**			
No	1	1	1
Yes	2.00 (1.60, 2.51)	3.00 (1.87, 4.81)	1.44 (1.00, 2.07)
**Ever had sex**			
No	1	1	1
Yes	1.57 (1.18, 2.08)	1.53 (0.99, 2.37)	1.75 (1.12, 2.72)
**Ever been physically attacked**			
No	1	1	1
Yes	1.38 (1.08, 1.75)	1.56 (1.06, 2.32)	1.53 (1.04, 2.26)
**Ever engaged in physical fight**			
No	1	1	1
Yes	0.96 (0.74, 1.25)	1.01 (0.69, 1.47)	0.80 (0.50, 1.29)
**Ever been bullied**			
No	1	1	1
Yes	2.69 (1.98, 3.63)	2.52 (1.61, 3.94)	2.84 (1.67,4.83)
**Ever missed class or school**			
No	1	1	1
Yes	1.34 (1.07, 1.67)	1.21 (0.80, 1.82)	1.41 (1.01, 1.97)
**Parent/guardian ever checked your homework**			
			
No	1	1	1
Yes	1.19 (0.95, 1.50)	1.54 (1.05, 2.26)	0.94 (0.66, 1.34)
**Parent/guardian ever understood your problems**			
			
No	1	1	1
Yes	1.18 (0.93, 1.48)	0.67 (0.46, 1.00)	1.15 (0.80, 1.65)
**Parent/guardian ever knew what you do on free time**			
			
No	1	1	1
Yes	0.82 (0.65, 1.04)	1.10 (0.75, 1.61)	0.62 (0.42, 0.91)

AOR; Adjusted Odds Ratio

* 10-19 years are in survey 2; 20-24 years, reference category for survey 2; 10-14 years, reference category for overall surveys

Overall, students aged 20-24 years had 4.4 times higher odds of mental health problems (AOR=4.41, 95% CI 1.96-9.90) compared to those aged 10-14 years. Higher odds of mental health problems were also among females (AOR=1.40, 95% CI 1.12-1.74), currently using any substances (AOR=2.00, 95% CI 1.60-2.51), ever had sex (AOR=1.57, 95% CI 1.18-2.08), ever been physically attacked (AOR=1.38, 95% CI 1.08-1.75), ever been bullied (AOR=2.69, 95% CI 1.98-3.63), ever missed class or school (AOR=1.34, 95% CI 1.07-1.67) compared to their counterparts.

## Discussion

This study aimed to determine the prevalence of mental health problems, compare the prevalence across two survey years, and determine associated factors among secondary school students 10-24 years in the Kilimanjaro region. The overall prevalence of mental health problems was 29.2%. Prevalence was highest in survey 2 (32.6%) than in survey 1 (27.4%). About 11.2% ever felt worried, ever felt lonely (8.5%), considered suicide (6.9%), planned suicide (4.5%), and attempted suicide (3.5%). Age, sex, survey year 2, current substance use, ever had sex, ever physically attacked, ever been bullied, and ever missed class or school were associated with higher odds of mental health problems.

The overall prevalence of mental health problems among secondary school students in the Kilimanjaro region was high in this study, with about 30% reporting at least one mental health problem in the past 12 months preceding the survey. This burden is alarming, and when not addressed, there will be more mental health problems in the future, which may have associated consequences such as suicide and school dropout. The burden was slightly higher in survey 2, which comprised older students (form fours) compared to survey 1 which had younger form one students. This is possible because form four students might have had national examination stress. This prevalence is lower than that reported among secondary school students in Dar es Salaam (41%)^20^. This is probably because of the sample size and geographical location differences between these studies. This overall prevalence is higher than the school health survey in Ghana (13.5%)^19^. In the present study, worry or anxiety appears more prevalent (11.3%) than other mental health behaviors, which is different from the 2014 Tanzania GSHS report (6.4%)^7^. The reason for being worried may be due to fear of failure in final examinations for those students in form four and fear of not fitting in the new environment for the form one students. From our results, the prevalence among form four students was 14.7% compared to 9.4% among form one students. The presence of form four students in our study affects our results given that in 2019 when students were in form one, they had less worries compared to when preparing for final ordinary-level exams in 2022. Students who felt lonely were 8.5% like the 2014 Tanzania GSHS report (7%)^7^. Students had sleep problems in both surveys. Similar estimates (5.1%) have been reported in a study in Tanzania^1^. Sleep problems have also been reported to be associated with suicidal behaviors, loneliness, and serious injuries^19^. Adolescents and young people with sleep problems may have nightmares that cause them to hurt themselves leading to suicide death^19^.

Suicidal behaviors were high in this study. On all suicide measures, there was an increase in prevalence with the survey year as older students (most of which were in survey 2) reported higher suicidal rates compared to younger students^17^,^21^. This may be due to school problems such as exam stress, social relationships, being lonely, worried, or bullied^17^,^22^. Much higher suicidal behaviors (ideation, plan, attempt) have been reported in the 2014 Tanzania GSHS survey^7^. The estimate in this study is like a study conducted in South Africa where 32.5% felt sad, 19% made a suicide plan, and 21.8% attempted suicide^23^,^24^.

Older students (20-24 years compared to 10-14 years) were more likely to have mental health problems as reported in South Africa^23^, Ghana^19^, England^10^, and China^11^. These results may be attributed to differences in survey years. During survey 2 (form four) when students are in late adolescence and young adult stage, they undergo psychological and social transition, which may affect their well-being both in the present and future life, as mental health problems when not treated persist to adulthood^10^. In addition, female students had higher odds of mental health problems compared to male students as reported elsewhere^7^,^13^,^19^,^25^. This observation has been attributed to physical, emotional, and social changes; for example, stressful life events such as menstruation and exposure to unwanted sexual activities^18^. Schools should put forward improved gender-based programs to help students, especially females whenever they experience stressful life events.

Students currently using any substances were more likely to have mental health problems. This was also reported in the Tanzanian National Adolescent Health and Development Strategy 2018-2022^1^. Having mental health problems has also been associated with history of having sexual intercourse especially among females in other settings^23^-^24^. The use of substances and engaging in sexual activities are used as depression and anxiety coping mechanisms, which lead to school dropout, and increased risk of sexually transmitted infections^12^. Students who missed classes or school had higher odds of mental health problems. This results to poor school attendance hence poor education achievement consequently impacting students' future. In addition, being bullied frequently during late adolescents (15-19) and among young people (20-24) was related to worry and loneliness. This leads to traumatic experiences, feelings, and behaviors that may lead to mental health problems in adulthood. Bullying has also a greater impact on adolescent health, especially on girls^26^. This happens because most schools in sub-Saharan African countries do not have programs that reduce bullying by educating adolescents and young people on different types of bullying and their effects on the victims^27^.

### Study limitations

This survey included only school-going adolescents, hence the findings cannot be generalized to all adolescents in Tanzania. The measure used for mental health problems in this study may be exaggerated and also over-estimate the prevalence. The repeated cross-sectional design of the study could have resulted in duplicate responses, bias, and unaccounted overlap because the participants in the two surveys were not linked. This may affect the statistical power, representativeness, and generalizability of the findings. As a result, caution must be used when interpreting the study's findings.

## Conclusion

The present study suggests that mental health problems are highly prevalent (29.2%) among secondary school adolescents and young people in the Kilimanjaro region relative to Tanzania's 2014 GSHS report (13%). Young people aged 20-24 years, females, current substance use, with a history of having sex, those ever been physically attacked, ever missed class, and who have ever been bullied were more likely to have mental health problems. Schools should improve programs that promote mental health awareness through physical activities such as sports, or meditation to address mental health issues. Students need the necessary resources and support such as mental health clubs, and counseling from psychologists or teachers, or peer counselors in the school environment.

## Figures and Tables

**Figure 1:**
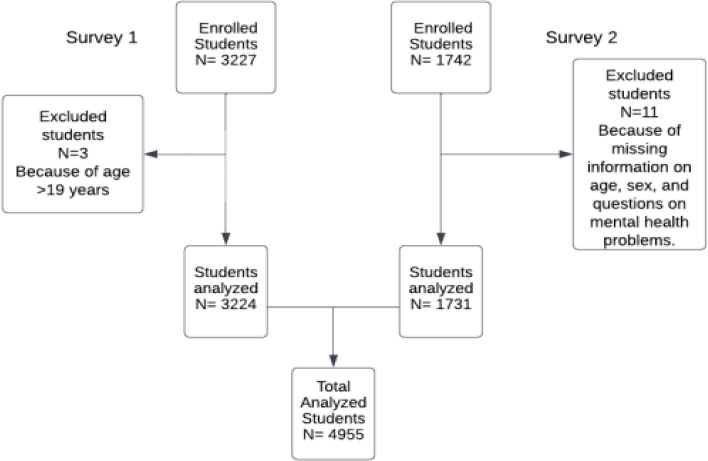
A flow diagram of enrolled and analyzed participants in the two surveys
